# The Insulin-like Growth Factor System and Colorectal Cancer

**DOI:** 10.3390/life12081274

**Published:** 2022-08-20

**Authors:** Nikola Gligorijević, Zorana Dobrijević, Miloš Šunderić, Dragana Robajac, Danilo Četić, Ana Penezić, Goran Miljuš, Olgica Nedić

**Affiliations:** Institute for the Application of Nuclear Energy, Department for Metabolism, University of Belgrade, Banatska 31b, 11080 Belgrade, Serbia

**Keywords:** colorectal cancer, insulin-like growth factor system, signaling, therapy, genetic regulation

## Abstract

Insulin-like growth factors (IGFs) are peptides which exert mitogenic, endocrine and cytokine activities. Together with their receptors, binding proteins and associated molecules, they participate in numerous pathophysiological processes, including cancer development. Colorectal cancer (CRC) is a disease with high incidence and mortality rates worldwide, whose etiology usually represents a combination of the environmental and genetic factors. IGFs are most often increased in CRC, enabling excessive autocrine/paracrine stimulation of the cell growth. Overexpression or increased activation/accessibility of IGF receptors is a coinciding step which transmits IGF-related signals. A number of molecules and biochemical mechanisms exert modulatory effects shaping the final outcome of the IGF-stimulated processes, frequently leading to neoplastic transformation in the case of irreparable disbalance. The IGF system and related molecules and pathways which participate in the development of CRC are the focus of this review.

## 1. Introduction

Insulin-like growth factors (IGFs), mitogenic and metabolic peptides, are involved in the etiology and progression of the colorectal cancer (CRC). The association was confirmed at the level of cell lines, animal models and patients with CRC. Initial studies on the role of IGFs, their receptors (IGFRs), high-affinity binding proteins (IGFBPs), IGFBP proteases, highly related insulin and its receptor (IR) expanded to investigate connections with an array of physiological molecules and systems which together build a very complex network not yet fully defined and characterized. Current research includes IGFBP-related proteins (IGFBP-RP, also known as low-affinity IGFBPs), messenger RNA (mRNA), responsible genes, circular-, micro- and long non-coding RNAs (circRNA, miRNA and lncRNA), RNA-binding proteins, other protein and nucleic acid binding partners inside specific compartments of cells and membrane-bound, present in the extracellular matrix and in the circulation. There are many pathways of interference, with the effects primarily based on the activation of the IGF system.

IGFs are known to exert endocrine, paracrine and autocrine effects. Many cell types synthesize IGFs, IGFRs and IGFBPs. The response of cells to IGFs depends on various factors, many of them being specific for the particular microenvironment at a specific pathophysiological moment. Modulatory effects of different agents have been detected, positive and negative, both at the expression level and on the activity of individual components of the IGF system. Tight control mechanisms are necessary to maintain the equilibrium in the normal physiological state since overexpression or excessive activation/accessibility of some components, together with a reduced activity of suppressor molecules, can lead to disbalance and neoplastic transformation. In this review, we focus on the IGF system and related molecules/mechanisms that participate in the initiation and the development of CRC.

## 2. Epidemiology of Colorectal Cancer

The world’s cancer burden represents one of the biggest hurdles for human life improvement [1]. According to estimates made by the World Health Organization (WHO) in 2019 [2], cancer is the primary or secondary cause of death of people below the age of 70 in 112 out of 183 countries, and the third or fourth cause in 23 additional countries [3].

**Figure 1:**
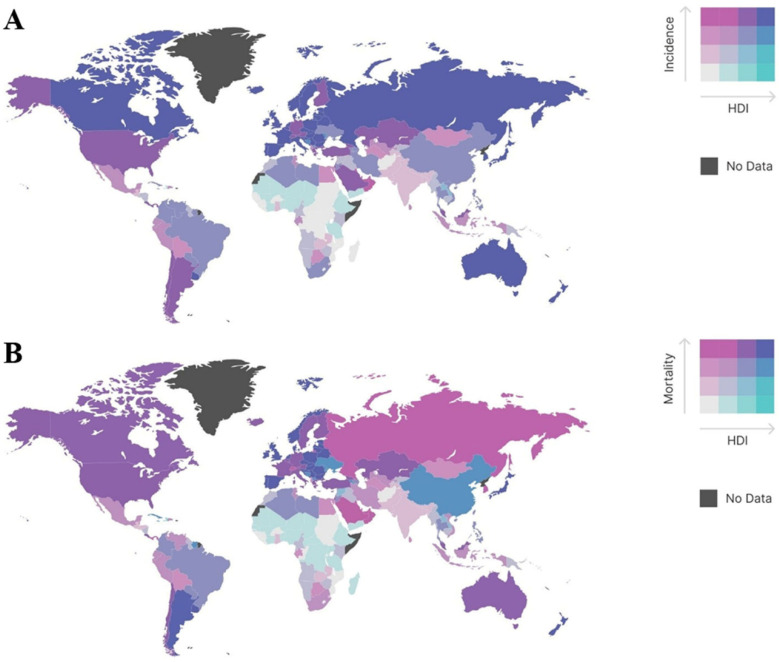
Global bivariate maps representing the link between human development index (HDI) and colorectal cancer incidence (**A**) or mortality (**B**). The boundaries expressed on this map do not imply the expression of any opinion whatsoever concerning the legal status of any country, territory, city or area, or of its authorities, or concerning the delimitation of its frontiers or boundaries. Data source: WHO, UNDP [2,6].

Colorectal cancer is highly ranked for its incidence and mortality—it is the third most frequently occurring malignancy, responsible for 10.0% of all cancer cases for both sexes, and it was the second most fatal cancer in 2018, with 9.4% of reported deaths [3]. More than 1.9 million new CRC cases occurred in 2020, resulting in 935,000 new deaths globally [3]. Complications, mortality, treatment side effects, health care service utilization and medical costs associated with CRC present a considerable burden worldwide. There are, however, significant geographical differences in CRC incidence and mortality. The age-standardized rate (ASR) of CRC incidence was found to be six times higher in high human development index (HDI) countries compared to low HDI countries, with a similar ratio found for ASR of mortality (Figure 1) [4,5,6].

Categorization of CRC cases can be performed by age—patients less than 50 years old belong to an early-onset CRC category, whereas older patients belong to a late-onset CRC category. In the late-onset CRC, HDI and EAPC (estimated annual percentage change) are negatively correlated for both sexes, implying that present lower incidence in low HDI countries might worsen in the next 30 to 50 years, posing a more serious health issue [5].

Men are more prone to developing CRC than women. Even though there is no discernable difference in the early onset of colon cancer between sexes (IRR = 0.98; 95% CI 0.94, 1.07), the incidence of early-onset rectal cancer (IRR = 1.08; 95% CI 1.04, 1.12), late-onset colon cancer (IRR = 1.50; 95% CI 1.37, 1.62) and late-onset rectal cancer (IRR = 2.03; 95% CI 1.84, 2.21) is considerably higher in males [5].

Smaller scale (i.e., continent- or region-specific) studies confirmed the correlation between an increased CRC incidence and high HDI. Chung et al. [7] reported such findings for Asia, while Sierra et al. [8] corroborated the impact of HDI on CRC in Central and South America.

Worldwide CRC incidence and mortality have generally increased in the 21st century and will continue to rise in the future, as estimated by numerous global [4,5,9,10], continental [7,8,11,12], regional [13,14,15,16,17] and country-specific [18,19,20,21] studies. Such trends warrant new and more effective screening and prevention strategies to be developed from evidence-based research.

## 3. Pathophysiology of the Colorectal Cancer

**Figure 2:**
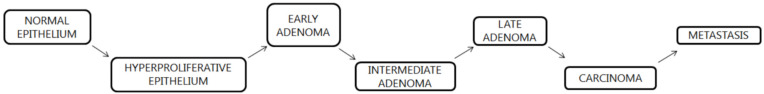
Multiple-stage neoplastic transformation of the colon tissue.

The etiology of CRC is multifactorial and usually represents a combination of the environmental and genetic factors. The process of neoplastic transformation consists of a series of (epi)genetic alterations that lead to changes in normal mucosa of the colon, resulting in cancer, which has the potential to culminate in metastasis in distant tissues [22]. Colon modifications can be divided into discrete stages which tissue passes, as proposed by Fearon and Vogelstein (Figure 2). Each stage is characterized by specific shifts in the genetic make-up of the cell [23].

There are several mechanisms that lead to neoplastic changes in colonocytes. The most common is the chromosomal instability (CIN) pathway, which is characterized by the loss of heterozygosity and various chromosomal abnormalities [24]. One of the first changes that occurs in this mechanism is dysregulation in the WNT/APC/β-cat pathway, responsible for the expression of *APC* gene, a tumor suppressor, leading to an increased presence of β-catenin, which induces proliferation, differentiation, migration and adhesion of colorectal cells [25,26]. Other changes follow, accompanied by new mutations and progression from benign to malignant state. The process includes mutations in *KRAS* and *p53* genes and their binding partners [27,28,29,30,31]. Another cause of CRC, which roughly accounts for 15% of cases, is a derangement in the DNA mismatch repair system (MMR), which is in charge of the production of proteins that recognize and repair single nucleotide mismatches arising in the replication process [32,33]. The third mechanism leading to CRC is based on hypermethylation of CpG islands in promoter regions of genes involved in cell cycle regulation, apoptosis, adhesion and invasion [34,35,36].

Chronic inflammation plays a pivotal role in disease etiology. Various inflammatory markers, such as tumor necrosis factor-α (TNF-α), signal transducer and activator of transcription 3 (STAT3), interleukin-6 (IL-6) and C-reactive protein (CRP), are associated with pathogenesis of CRC. TNF-α promotes tumor growth, proliferation and metastasis. IL-6 stimulates the expression of STAT3, which is a transcription factor that induces the expression of various genes that play active roles in cell proliferation, differentiation and apoptosis, such as *Bcl-2*, *CyclinD1*, *ICAM-1* and *MMP2-9* [37,38,39]. Cyclooxygenase-2 (COX-2) is an inducible cyclooxygenase that is up-regulated by cytokines, growth factors and tumor promoters. It is overexpressed in 40% of human colorectal adenomas, compared to normal epithelial tissue [40]. COX-2 regulates prostaglandin (PG) synthesis, apoptosis, angiogenesis and tumor invasiveness, being a mediator between the inflammation and neoplastic transformation in CRC.

## 4. Insulin-like Growth Factors and Colorectal Cancer

### 4.1. Insulin-like Growth Factors

The IGF system is composed of two IGF peptides (IGF-I and IGF-II), two receptor types (IGF1R and IGF2R) and six high-affinity IGF-binding proteins (IGFBP-1 to -6) [41]. The first information on the so-called somatomedins, now known as IGFs, appeared in the 1950s [42]. Many cell types synthesize IGFs, but IGFs in circulation are mostly derived from the liver [41]. IGF-I synthesis in the liver is under the control of the growth hormone (GH), whereas the production of IGF-II is less GH-dependent. IGF-II is the major growth factor during fetal development. During adult life, its role seems to be of less significance compared to IGF-I. The importance of IGF-II increases in tumor development, as some tumors, including those of the colon and rectum, produce high concentrations enabling autocrine/paracrine stimulation of the cell growth [41,43]. Not only the fully processed IGF-II, but also the so-called high molecular weight IGF-II pro-peptides can exert growth-promoting effects in tumors, as well as general hypoglycemia. Non-islet cell tumor hypoglycemia in CRC patient is a rare event [44].

IGF-I regulates growth and development during post-natal life, and its circulating concentration declines with age after puberty and depends on lifestyle and nutritional habits [41]. Circulating and local concentrations of IGF-I and IGF-II often do not correlate, as was shown for the serum and colorectal tissues [45]. The expression level of IGF-I mRNA, and particularly IGF-II mRNA, is several times greater in colon cancer specimens than the actual concentration of proteins [46]. In some cases, a positive correlation was found between tumor grade and IGF-II expression. Paracrine and autocrine mechanisms of IGF-II action in colon cancer were documented, as cancer-associated fibroblasts in tumor stroma were identified as a source of up-regulated IGF-II which, in turn, activates pro-survival IGF1R/IR signaling [47].

### 4.2. Insulin-like Growth Factor Receptors

Receptor IGF1R is a tyrosine kinase which becomes activated after binding of IGF-I or IGF-II, inducing its auto-phosphorylation and a signaling pathway which will end up primarily in the growth-promoting events [41,48]. Phosphorylated receptor substrates are a common signaling meeting point of several receptor systems including those for other growth factors, integrins, cytokines [49]. IGF1R is widely expressed in the gastrointestinal tract, the most in the colonic crypt proliferating cells [50].

Insulin and insulin receptor (IR) are tightly connected to the IGF system, as IGFs exert some of their functions (primarily metabolic) after binding to IR. Structural homology between IGF1R and IR enables formation of the hybrid receptor (HyR) consisting of IGF1R and IR hemi-receptors, capable of binding both insulin and IGFs [51]. IR exists in two isoforms, IR-A and IR-B, with the IR-A form mainly present in fetal and cancer tissues [52,53]. HyR containing IR-A is approximately two times more expressed in CRC tissue than in normal epithelial cells [54].

There is also functional homology between IGF1R, IR and HyR, as they all initiate cell signaling cascade, relying on the activation of insulin receptor substrates (IRS-1 to -6) [55]. The relative importance of the intracellular pathways that will serve as effectors of the signal transduction is dependent on the specific cell context and related to other intrinsic and external factors. Besides by synthesis, the availability of receptors is regulated by endocytosis and proteolysis [56].

Receptor IGF2R (also known as cation-independent mannose-6-phosphate receptor, CI-MPR) binds only IGF-II with high affinity [57]. This interaction most often leads to the removal of IGF-II and its degradation, but also some signaling events are mediated through this pathway. The extracellular domain of IGF2R can be found in a soluble form in physiological fluids exerting a role of the circulating IGF-II scavenger [58].

As previously mentioned, COX-2 and PGs play important roles in the development of CRC and the expression of COX-2 mRNA, and PGE2 is higher in intestinal cells which overexpress IGF-II, the effect being mediated via IGF1R [59]. Both IGF1R and IGF2R are present on the epithelial cells of the intestine. Overexpression of IGF1R is detected in CRC samples compared to adjacent normal mucosa [60,61]. A degree of IGF1R expression is correlated with an increased risk of metastasis in CRC patients with Dukes’ score C [62], but not with patient survival [63]. The glycosylation pattern of both IGF1R and IGF2R changes in colon tissue due to CRC [64], and receptors are also modified by oxidation [65]. These post-translational modifications possibly affect ligand binding, activation and/or other interactions of receptors.

When measuring the presence of specific components of the IGF system, one should be aware of the difference in their expression levels at different locations in healthy tissue. The expression of IGF-I, IGF-II and IGF1R mRNAs is significantly higher in rectum compared to ascending colon, while there is no difference in the expression of IGF2R mRNA [45]. Furthermore, the analysis of two samples from the same location obtained during the same biopsy procedure may differ due to different expression of individual members of the IGF system in different cell types, i.e., epithelial and stromal cells.

### 4.3. Cell Signaling via Insulin-like Growth Factor Receptors

**Figure 3:**
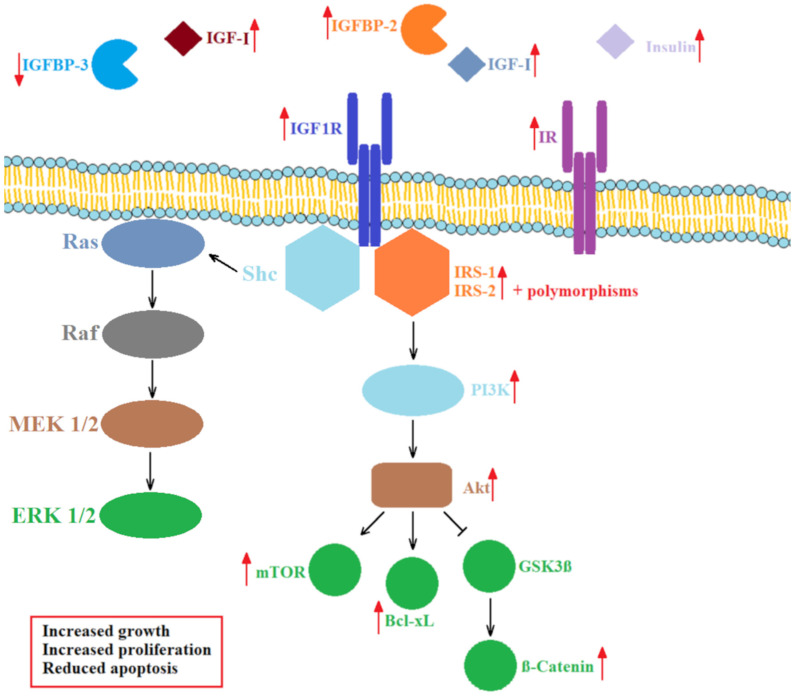
Schematic presentation of IGF1R/IR-dependent cell signaling pathways and molecules altered in CRC. Upward and downward red arrows indicate a direction of change. IR—insulin receptor; IGF—insulin-like growth factor; IGF1R—insulin-like growth factor receptor; IGFBP—IGF binding protein; IRS—insulin receptor substrate; PI3K—phosphoinositide 3-kinase; Akt—protein kinase B; GSK3β—glycogen synthase kinase 3-β; Bcl-xL—B-cell lymphoma extra-large; mTOR—mammalian target of rapamycin; Shc—Src homology and collagen adaptor protein; Ras—rat sarcoma virus-related protein; Raf—serine/threonine-specific protein kinase; MEK—mitogen-activated protein kinase; ERK—extracellular signal-regulated kinase.

Binding of IGFs to IGF1R initiates auto-phosphorylation of its intracellular tyrosine (Tyr) residues, docking of IRS and activation of intracellular kinases, such as mitogen-activated protein kinase (MAPK) and phosphoinositide 3-kinase (PI3K), inducing a phosphorylation cascade leading to growth-stimulating events [41,48]. IGF1R and IR signaling rely predominantly on the activation of IRS-1 and IRS-2 (Figure 3). The type of the substrate activated influences downstream signaling. IRS-1 expression seems to be inversely correlated with cell differentiation in CRC [66], whereas IRS-2 expression positively correlates with the transformation of the intestinal epithelium to adenocarcinoma [67]. Certain polymorphisms regarding these two proteins are associated with a risk of CRC [68]. An increase in phosphorylated IR was found to accompany transformation of normal colorectal epithelium to CRC, suggesting the involvement of IR in carcinogenesis [69].

Activated PI3K further stimulates the activation of Akt and the expression of B-cell lymphoma extra-large (Bcl-xL) protein, which regulates apoptosis, and the mammalian target of rapamycin (mTOR) signaling pathway, which regulates cell growth, proliferation, motility, survival and the general metabolism. Overexpression of IGF1R, Bcl-xL and mTOR inhibits apoptosis and contributes to colon cancer cell survival and invasion [70]. At the same time, activated Akt inhibits glycogen synthase kinase 3-β (GSK3β), causing translocation of β-catenin to the nucleus and transcription of genes involved in cell proliferation [71]. IGF1R-dependent pathways also play a significant role in the resistance to therapeutic agents, which we examine in the last section of this review.

IGF-I signaling was identified as a contributing factor in the Warburg effect, increased glucose uptake and a metabolic switch from butyrate oxidation to aerobic glycolysis, which is one of the hallmarks of CRC [72]. Insulin/IGF signaling was shown to induce the expression of hypoxia-inducible factor 1α (HIF-1α), which is the main regulator of Warburg effect [73]. Activation of both IGF1R-related pathways, PI3K/Akt/mTOR and Raf/MAPK, was found to be associated with glycolytic metabolism in CRC [72]. Furthermore, the relation between these signaling pathways and glucose metabolic reprograming in CRC could be partially attributed to the activities of lncRNA CRNDE [74,75].

### 4.4. Insulin-like Growth Factor Binding Proteins

IGF-binding proteins are synthetized in different cell types. They form complexes with IGFs which may reside within blood vessels (mostly tertiary complexes, maintaining the IGF reservoir) or they may cross the endothelial barrier to transport IGFs to peripheral tissues (mostly binary complexes) [41]. The most prevalent IGFBPs in the circulation, namely, IGFBP-3, IGFBP-2 and IGFBP-1, are derived from the liver. Some IGFBPs are synthetized locally where they bind IGFs and regulate their presentation to receptors. IGFBPs can exert either inhibitory or potentiating effect on IGFs, and their affinity for IGFs can be altered by post-translational modifications and interactions with other proteins, cells or extracellular matrix [76]. IGFs need to be in a free form in order to interact with receptors and IGFBP proteases enable their liberation [76]. IGFBPs cleaved by proteolysis have reduced affinity for ligands facilitating IGFR capture of IGFs.

IGFBP-3 is the major IGF-binding protein. Approximately 85% of the circulating IGFs are bound to it, together with an acid-labile subunit [77]. Investigations on cancer risk take into account not only the concentration of IGFs and IGFBPs, but particularly the ratio of IGFs and IGFBP-3. An unfavorable ratio was found in patients with CRC [78]. Although an increased ratio IGF-I/IGFBP-3 was related to the initiation of CRC, the relation could not be clearly established with the progression or the outcome of CRC. Most studies report on the protective role of IGFBP-3 in CRC [79,80], but there are also contrasting results [81,82]. Higher expression level of IGFBP-3 mRNA was detected in normal colon than in cancer. In contrast to normal colon, where this mRNA was distributed both in epithelial tissue and stroma, it is mainly present in a stromal component of the cancer tissue, suggesting its paracrine role [82]. A correlation between the expression of mRNA IGFBP-3, disease staging or tumor location in the colon was reported [83]. High expression of IGFBP-3 mRNA correlates with lymph node metastasis and poor outcome. An increased sialylation of IGFBP-3 originating from patients with CRC was also detected [84].

Colon cancer cell lines most often secrete IGFBP-2 and it is frequently overexpressed in CRC tissues, especially glandular [60]. According to some studies, an increased circulating concentration of IGFBP-2 is associated with CRC and in correlation with the advancement of a disease [60,85,86]. According to the others, IGFBP-1 and IGFBP-2 concentrations in blood are not associated with colon or rectal cancer [77,87,88].

IGFBP-4, which exerts only inhibitory action on IGFs, is often overexpressed in cancer and in correlation with a state of differentiation. IGFBP-4 was shown to inhibit growth of some colon cancer cells [89]. Stimulation of Caco-2 cells with IL-1β and IL-6 was shown to modulate secretion of IGFBP-2 and IGFBP-4 [90], confirming a connection between inflammation and carcinogenesis. IGFBP-6 is supposed to act as tumor suppressor and its concentration is lower in metastatic compared to non-metastatic CRC cells [91]. Furthermore, IGFBP-6 expression in CRC tissue is inversely correlated with the survival of patients [92] and connected to the tumor suppressor activity of SEMA3B [93].

IGFBPs exert IGF-dependent and IGF-independent roles. Some IGFBPs bind to specific receptors on the cell surface and/or to nuclear receptors [89,94]. IGF-independent actions of IGFBP-3 in CRC are primarily connected to its pro-apoptotic role through NF-κB inhibition [95], interaction with retinoid X receptor (RXR)/Nur77 [96] or p53-dependent signaling cascade [97]. No clear IGF-independent action of IGFBP-1 was observed in CRC [98], although increased levels of IGFBP-1 and decreased incidence of CRC seem to be linked in women [99]. Elevated serum level of IGFBP-2 was nominated as a potential biomarker of CRC [85]. However, an increased expression of IGFBP-2 mRNA in colon cancer cells and tissue is accompanied by an increased proteolytic degradation [100], calling for further investigations to define its potential local role in CRC. IGF-independent actions of IGFBP-6 are mainly tumor protective and are considered as possible paths for cancer therapy [101]. The same authors also reported a promotion of LIM 1215 colon cancer cell migration stimulated by IGFBP-6 [102], which leaves the final conclusion on the role of IGFBP-6 in colon cancer still to be assessed.

Some data concerning IGFBP concentrations and involvement in CRC (and in other malignancies as well) seem controversial due to the complexity of the IGF system, its relations with other physiological molecules and a variety of signaling mechanisms. Age, BMI, gender, physical activity, nutritional habits and possibly ethnicity contribute to disagreements reported in different studies [103]. Obesity-related insulin/IGF signaling pathways influence CRC development by evading apoptosis, whereas reported effects of exercise are inconsistent and without clear conclusions [104,105]. Maintenance of glucose control via nutritional habits has a major influence on the insulin/IGF axis in relation to CRC [106].

IGFBP-7, the most studied member of the IGFBP-RP family, shares certain signaling pathways with IGFBP-3 [107]. According to some studies, it is up-regulated in colon cancer, but according to others, it is down-regulated. Contradictory results were reported both in patients and colon cancer cell lines. Some results suggest IGFBP-3/IGFBP-7 interaction, but this crosstalk needs additional confirmation. The association seems to participate in cancer progression and metastasis [108].

The putative roles of IGFBPs are summarized in Table 1.

Due to the complexity of CRC etiology as well as the complex role of the IGF system in CRC pathology, it is hard to distinguish mechanisms of IGFBP regulation which can be directly linked to the incidence of CRC from those primarily involved in further development of cancer. The choice of the study group has a significant influence on the results. A study conducted on older men from Australia suggested that higher IGFBP-3 concentrations are associated with an increased incidence of CRC [109]. In a study on the general population in China (both men and women), the initiation of CRC was associated with an increased ratio IGF-I/IGFBP-3 as well as reduced IGFBP-3 concentration [78]. As for IGFBP-2 concentration, the data are controversial, since some suggest association with CRC incidence, while others fail to confirm the link. In IGFBP-2 transgenic mice which overexpress IGFBP-2, CRC incidence was similar to that of the control group of animals, but the volume of adenomas was more than two times smaller [110]. Although it is accepted that IGFBP-4 inhibits the action of IGF-I, and thus, CRC progression, very little is known about its role in CRC incidence. IGFBP-4 gene therapy could not prevent CRC in mice inoculated with HT-29 colon cancer cells, but an increased apoptosis was detected [111]. In a case-controlled study conducted on women from New York, lower concentrations of IGFBP-1 were linked to a decreased incidence of CRC [99]. The same study also demonstrated that women in a quintile with the highest IGFBP-3 concentration were at higher risk for CRC development, compared to those in a quintile with the lowest IGFBP-3 concentration.

### 4.5. Insulin-like Growth Factor Binding Protein Proteinases

Matrix metalloproteinases (MMPs) regulate remodeling of the extracellular matrix (ECM), which is an important event for metastatic progression of tumor, and some of them also act as IGFBP proteases, ensuring a simultaneous release of IGFs to stimulate IGF1R signaling. MMP-7, often increased in malignant diseases, is capable of degrading all six IGFBPs [112]. CRC cells which overexpress MMP-7 resist chemotherapy due to intensive IGFBP-3 degradation and release of IGFs [113]. MMP-19 is another proteinase which degrades IGFBP-3 in CRC [114]. IGF1R activation was identified as a promoting factor in the synthesis of MMPs.

Other MMPs, such as ADAM17 and DAM28, specifically cleave IGFBP-3 and increase IGF-I availability in CRC [115]. Pregnancy-associated protein A is IGFBP-4 specific proteinase and is expressed in the colon tissue [89]. Less IGFBP-4 is found in the cancer tissue than in the normal. Cathepsins, plasmin, kallikreins, pepsin, calpain and caspase also act as IGFBP proteinases [81,91,116,117] and are increased in CRC. An interplay between IGFs, IGF1R, IGFBPs and IGFBP proteinases plus other modifying factors define a response of the cell, including its (un)responsiveness to potential tumor-suppressive agents [48,118].

### 4.6. Genetic, Epigenetic and Post-Transcriptional Regulators of the Insulin-like Growth Factor System

#### 4.6.1. Genetic Variants

Profiling of genetic alterations in various components of the insulin/IGF system in CRC tissue and cell lines demonstrated a whole spectrum of mutations which can contribute to carcinogenesis. Common mutations in genes encoding downstream members of the insulin/IGF pathway, such as *KRAS* and *BRAF*, are known predictors of the biological potential of CRC and also dictate a response to treatment relying on IR/IGF1R inhibitors [119]. Another type of canonically driven mutations in CRC, affecting *TP53*, alters insulin/IGF signaling due to an interplay between p53/MDM2 and IGF1R-activated pathways [120]. Mutations in *PIK3CA* are observed in 20–25% of patients with CRC, suggesting potential application of IGF1R-targeting immunotherapeutic approaches [121]. Additionally, a loss of expression of *PTEN*, an important negative regulator of the PI3K/AKT signaling pathway, characterizes a large portion of CRC cases and is partially caused by mutational events [122].

Apart from somatic mutations, inherited germline variants in the insulin/IGF pathway genes were investigated in the context of CRC carcinogenesis. However, associations of these candidate variants with CRC susceptibility were found in a relatively small number of studies and were rarely replicated. Reports include both potentially functional and tagging variants within *IGF-I* (rs12423791, rs1019731, rs5742632, rs5742678, rs5742694, rs2033178, rs2373722, rs1520220, rs10735380, rs6220, rs6214, rs35767, 19-CA repeat polymorphism), *IR* (rs10426094, rs1052371), *IGFBP-3* (rs2854744, rs2854746, rs3110697, rs2132572, rs35440925), *IGF1R* (rs2229765, rs7166348), IRS-1 (rs1801278) and IRS-2 (rs1805097, rs2289046, rs754204, rs4773082) [68,123,124,125,126,127,128,129,130,131,132,133,134,135,136,137,138,139].

#### 4.6.2. DNA Methylation and Imprinting

Epigenetic mechanisms were determined as a cause of dysregulated expression of several IGF-related genes abundantly occurring in CRC patients. For instance, *IGFBP-3* promoter region is commonly hypermethylated in CRC, representing potential diagnostic and predictive biomarker [140,141]. A reduced expression of *PTEN* is partially caused by hypermethylation, which is frequent in CRC with microsatellite instability [122,142]. On the other hand, a genome-wide DNA methylation analysis demonstrated hypomethylation of more than 30 genes involved in PI3K/AKT signaling [143].

*IGF-II* was the first gene found to be paternally expressed due to genomic imprinting, since differentially methylated region (DMR) is methylated on the maternal allele [144,145]. An aberrant imprinting *IGF-II* locus was detected in CRC, leading to an overexpression of *IGF-II* and, subsequently, to higher proliferation rate and invasiveness [146,147,148,149]. The same imprinted region also contains a gene encoding H19, a long non-coding RNA (lncRNA) with a known regulatory role in the molecular basis of CRC carcinogenesis [150,151].

#### 4.6.3. Regulatory RNAs

MicroRNAs (miR) are important regulators of genes involved in the insulin/IGF signaling with their oncogenic or tumor-suppressive properties in CRC and in malignancy in general. For instance, the expression of *IRS-1* is regulated by miR-145, a commonly deregulated microRNA in CRC [152,153]. The same gene was also identified as a target of tumor-suppressive miR-497 and miR-126 [154,155]. MiR-143, originating from the same precursor as miR-145, as well as members of the let-7 family, miR-497, miR-184 and miR-98, were identified as regulators of *IGF1R* in CRC [156,157,158,159,160]. Common tumor-suppressive microRNA, miR-375, directly targets PI3K (PIK3CA) in CRC, while miR-92 exhibits oncogenic activity through the inhibition of a set of tumor-suppressors, including *PTEN* [161,162]. Similarly, miR-21 and miR-26a target *PTEN*, but lead to enhanced PI3K/AKT signaling [163,164]. Additional IGF-related targets of miR-143 include components of the RAS/RAF/MAPK pathway, highlighting the importance of tumor-suppressive activities of this microRNA in the molecular basis of CRC [153,165,166]. Various genetic variants within the *miR-143/145* locus were found to be associated with CRC susceptibility, risk of developing other malignancies and cancer aggressiveness [167,168,169,170,171,172,173,174,175,176].

Overexpression of an oncogenic lncRNA, colorectal neoplasia differentially expressed (CRNDE), is triggered in early stages of carcinogenesis in colorectal tissue [74]. Multiple splice variants are generated and the resulting isoforms have different intracellular localizations, which underlies their functional differences. Nuclear isoforms contribute to the Warburg effect in CRC by modulating transcription and acting as regulators of glucose and lipid metabolism. The expression of splice variants with retained introns is strongly regulated by insulin and IGF-I, as these variants are downstream targets of both PI3K/Akt/mTOR and RAF/MAPK signaling cascades. Furthermore, CRNDE nuclear transcripts regulate insulin/IGF signaling by a feedback mechanism [74]. Another mechanism connecting oncogenic properties of CRNDE with insulin/IGF signaling refers to sponging of microRNAs known to regulate the expression of key components of this signaling cascade, such as *IRS-1* [177]. An increased expression of CRNDE was found both in tumor tissue and in plasma from CRC patients, qualifying this lncRNA as a potential circulatory biomarker of CRC [178].

Another lncRNA potentially involved in CRC pathogenesis is PTENP1, a homologue of tumor-suppressive gene *PTEN* [179]. By acting as a microRNA sponge, *PTENP1* regulates the expression of PTEN. A similar mechanism is proposed for *KRAS1P* transcript, which regulates KRAS/RAF/MAPK signaling cascade [179]. PVT1, another lncRNA up-regulated in CRC, functions as an indirect regulator of IRS-1 expression through interaction with miR-214-3p [180].

#### 4.6.4. RNA-Binding Proteins

RNA-binding proteins (RNBs), together with mRNAs, ribosome subunits and other proteins, form ribonucleoproteins which function as RNA regulators. RNBs, such as IGF-II mRNA binding proteins (IGF2BPs), can be differentially expressed in cancer. IGF2BP family consists of three members which exert negative effects in the late phase of embryo development; their expression is lost in adults and may be reactivated in tumors [181,182]. IGF2BP1 was evaluated as an independent prognostic marker in CRC associated with the responsiveness to chemotherapy [183]. Although IGF2BP expression is generally related to worse prognosis, opposing overall effects of different IGF2BP members in different cells were detected [48].

### 4.7. Insulin-like Growth Factor Signaling and the Associated Signaling Pathways

A complex and interrelated network of events governs growth and spreading of cancer cells, with the IGF system being both modulated by other factors and exerting modulation of signals driven by a variety of other agents. This section briefly overviews other signaling pathways which interfere with IGF signaling.

Tumor growth depends on the oxygen supply and CRC is an example of a solid cancer where hypoxia emerges due to the inability of the vascular system to adequately supply oxygen to the growing tumor tissue [184]. IGF-I activity can induce the expression of HIF-1α, which may further trigger neovascularization in order to secure oxygen and nutrients for further tumor progression. Thus, hypoxia and activity of IGF-I are two parallel paths for the induction of HIF-1 in cancer [73], and the expression of HIF-1α and vascular endothelial growth factor (VEGF) in CRC tissues was proposed to serve as a biomarker of cancer progression [185]. Even more, hypoxia induces up-regulation of IGF synthesis through the STAT5b pathway [186], promoting tumor growth [187].

A crosstalk between IGF1R and several other membrane-associated molecules modifies IGF signaling. Cadherins are calcium-dependent transmembrane proteins which mediate cell–cell adhesion, whereas their intracellular domain participates in cell signaling. Cadherins mediate epithelial–mesenchymal transition (EMT), which is a crucial process in embryonic and tumor development. E-cadherin is down-regulated and N-cadherin is up-regulated during EMT [188]. IGF1R without IGF and E-cadherin in close proximity interact. This partnership is uncoupled upon IGF binding, as was shown in colon cancer cell lines [189]. Alpha V integrin is another binding partner creating a dynamic complex controlled by α-catenin, which may contribute to the cancer cell motility [189]. Involvement of α-catenin in IGF-I-induced cellular migration, but not invasion, in colon cancer cells was documented [190].

Discoidin domain receptor 1 (DDR1) is a tyrosine kinase which binds extracellular collagen, undergoes phosphorylation of the intracellular domain and participates in a signaling cascade leading to tumor progression [191]. Contrary to E-cadherin, IGF1R and DDR1 associate upon IGF binding, further stimulating the expression of IGF1R and downstream signaling [192]. DDR1 is expressed in colon mucosa and to a greater extent in cancer than in normal tissue. The association of its expression and tumor prognosis, however, awaits further evaluation [193].

Decorin is proteoglycan able to suppress the activity of some tyrosine kinases, including IGF1R [194,195]. A dichotomy of its activity on the IGF system was noted, depending on the cell type [196]. Decorin deficiency in Dcn-/- mice was shown to stimulate EMT and colon cancer metastasis [197].

IGFBP-3 and transferrin (Tf), an iron transporter and growth-promoting factor [198], form complexes which interact with Tf receptor (TfR). A reduced serum concentration of IGFBP-3/Tf complexes and an increased expression of TfR1 on colon cell membranes was found in patients with CRC [199]. IGFBP-1 and IGFBP-2 form complexes with alpha-2-macroglobulin (α2M) in the circulation [200,201]. The amount of IGFBP-2/α2M complexes is decreased in patients with CRC, although the total concentration of IGFBP-2 is increased [86]. Oxidative conditions modify the structure of IGFBP-binding partners, influencing the formation of complexes [202]. The exact role of these complexes is still unknown, but they contribute to the (re)distribution of IGFBPs between several molecular forms.

## 5. Therapeutic Potential of the Insulin-like Growth Factor Signaling Pathways in Treating Colorectal Cancer

As already said, neoplastic cell growth and proliferation is driven via PI3K, Akt, mTOR and MAPK pathways after IGF-I binding to IGF1R [203]. The primary strategy in treating colon cancer includes the arrest of IGF1R overexpression by small inhibitors, antibodies or the inhibition of its ligands. A discovery of OSI-906, later termed linsitinib, a drug with a promising inhibitory effect on two receptor kinases, IR and IGF1R, was reported in 2009 [204]. This dual inhibitor intervenes in the process of autophosphorylation, demonstrating an antiproliferative effect on different tumor cell lines, including colorectal. One possibility to treat metastatic CRC is to use regorafenib (multi-kinase inhibitor) together with linsitinib and aspirin (both being IGF1R inhibitors) [205]. Linsitinib and aspirin reduce the resistance of CRC cells to regorafenib, enabling body weight gain and an increase in the survival rate of model animals (i.e., male mice). Leiphrakpam et al. reported on the down-regulation of X-linked inhibitor of apoptosis (XIAP) in CRC xenografted male mice driven by MK-0646 (mAb that blocks IGF1R and IGF2R, also known as dalotuzumab) and linsitinib [206].

An interplay of various factors keeps cancer cells alive. Hyperactivation of IGF1R, for example, results in the resistance to epidermal growth factor receptor (EGFR) inhibition in RAS wild-type metastatic CRC via up-regulation of PI3K/AKT pathway, implying that targeting both receptors might be a promising therapy for metastatic CRC. Unfortunately, a combination of cetuximab (mAb targeting EGFR) and MK-0646 or IMC-A12 (mAb that blocks IGF1R, also known as cixutumumab) did not lead to the expected results, as suggested by two studies both involving male and female participants [207,208]. A phase I trial employing a combination of cixutumumab (anti-IGF1R antibody) and selumetinib (MEK ½ inhibitor) obtained promising results offering the evidence of the health benefit and target inhibition in a cohort of 30 patients, including those with CRC [209]. A combination of ganitumab (IGF1R mAb) and conatumumab (a pro-apoptotic death receptor 5 agonist) also exerted promising effects in the Colo-205 xenograft model, but caused no response in approximately 80 tested individuals (both male and female), some of whom were patients with CRC [210].

MEDI-573 (mAb to IGF-I and IGF-II) application inhibited tumor growth in a CRC female mouse model that over-expresses IGF-II, and this effect was enhanced if MEDI-573 was combined with other known therapeutics such as trastuzumab, AZD2014, AZD5363, selumetinib or cetuximab [211]. AvFc lectibody, a combination of the human immunoglobulin G1 Fc and Avaren (high mannose glycan-binding lectin), selectively recognizes a range of cancer cell lines including colon cells [212]. Although the observed cytotoxic effects were thoroughly investigated only in the lung cancer cell lines, it was confirmed that targets of this lectibody are EGFR and IGF1R. Since aberrant glycosylation is a hallmark of cancer, and altered glycosylation of IGFRs in the colon tissue of CRC patients was reported [64], the lectibody treatment may be considered as a novel approach in cancer therapy.

IGF1R depletion/inhibition sensitizes CRC cells to radiotherapy (converting them to radiosensitive), as shown in HT-29 and SW480 cell lines where IGF1R was inhibited by NVP-ADW742 [213] and in HT-29, SW480 and DLD-1 cells pretreated with BMS-754807 [214]. The inhibitory effect of BMS-754807 on colon cancer cell growth was stronger compared to the effect of linsitinib, and the anti-neoplastic effect was mostly independent of IGF1R [215]. A prolonged treatment of colon cancer cells with BMS-754807 and GSK1838705A (inhibitors of IGF1R and IR) leads to cell survival, due to the activation of ribosomal protein S6 kinase 1 [216]. In GEO tumors, BI885578 administration (inhibitor of both IGF1R and IR) induces apoptosis and inhibition of cell proliferation in female mice [217]. Zhu et al. reported on an anti-proliferative, anti-migration, anti-invasion and inhibitory effect of isovitexin on EMT in human colon cancer cell lines and xenograft tumor model on female mice [218]. The levels of signaling molecules involved in the IGF1R signaling pathway were decreased after this treatment, pointing to the mechanism of the isovitexin action. Simvastatin down-regulates IGF1R expression and pro-apoptotic ERK activation in human HT-29 cells [219]. Phloroglucinol treatment inhibits or decreases the expression of various IGF1R downstream signaling molecules, such as RAS, MAPK, ERK, PI3K, Akt and mTOR, in HT-29 cells [220].

In addition to synthetic molecules and antibodies, numerous plant metabolites were investigated as potential therapeutics in the treatment of cancer. Curcumol, isolated from *Rhizoma Curcumae*, inhibited proliferation and induced apoptosis in LoVo cells, and inhibited CRC in xenograft models of nude mice, via inhibition of IGF1R and activation of p38 MAPKs [221]. Application of manuka honey (alone or in combination with 5-fluorouracil) on HCT-116 cell line decreased physical parameters of colonospheres and the survival ability of cancer cells, but also induced apoptosis via down-regulation of apoptosis inhibitors, including IGFs and IGF1R [222]. The expression levels of IGF-I and IGF1R were reduced, whereas the level of IGFBP-3 was normalized after *Bifidiobacterium longum* BAA-999 (with or without lycopene) was administrated to CD-A male mice in an azoxymethane-dextrane sulfate sodium-induced CRC model [223]. Polypeptides from *Arca subcrenata* Lischke inhibited growth of HT-29 cells and suppressed tumor growth in male mouse xenograft by reducing IGF1R phosphorylation and inhibiting IGF-I/IGF1R signaling activation [224]. Carnosic acid treatment also suppressed the growth of HT-29 cells; decreased the number of tumors and circulating concentrations of leptin, adiponectin, insulin and IGF-I; and reduced the expression of IR in a male mice model [225]. Fucoidan, sulfated polysaccharide from brown algae, inhibited IGF1R signaling through the IRS-1/PI3K/Akt pathway in HT-29 cells [226]. Laminarin, another polysaccharide from brown algae, decreased phosphorylation of ERK and MAPK and, consequently, IGF1R-dependent proliferation in the same cells [227]. Curcumin decreased the expression of IR and IGF1R in 5-fluorouracil-treated SW480 cells, and this down-regulation correlated with decreased proliferation and migration of cells [228]. Luteolin, a fruit and vegetable flavone, decreased IGF-II production in HT-29 cells and down-regulated the activation of PI3K/Akt and ERK1/2 pathways [229]. Cinnamaldehyde, isolated from stem bark of *Cinnamomum cassia*, inhibited PI3K/Akt signaling in SW480, HCT116 and LoVo cells [230]. Considering the incidence and mortality rates of CRC, together with limitations of the existing therapeutic procedures, the search for both natural and synthetic anti-CRC agents is expected to intensify.

It is sometimes necessary to apply multiple approaches, i.e., a combination of different therapeutics and therapies. A serious limitation in examining therapeutic potentials of various substances can be found in translation from cell line models to xenografts and further to humans as the results are unsatisfactory or the cancer cells are nonresponsive to just one therapeutic. It must also be noted that the gender equality was neglected in most of the mentioned studies, as some of the experiments were performed only on male or female animals (rarely both). Furthermore, the number of studies on humans is still low, which is understandable due to the limited number of potentially effective therapeutics, their toxicity and effective dose, as well as ethical principles related to studies on humans.

## 6. Conclusions

The insulin-like growth factor system exerts multiple effects and at multiple levels in colon cancer transformation. The results obtained with tumor cells, animals and humans, however, show differences, some related to a tumor/cell type, but other factors (besides methodological) seem to be very influential. Caution is suggested with the generalization of effects, as crosstalk between the IGF system and other biochemical pathways regularly occurs and contributes to the final cell signaling event. Similarly, the complexity of interrelated (patho)physiological paths and outcomes contributes to the resistance to therapeutic approaches in CRC treatment.

## Figures and Tables

**Table 1:** Known or suspected roles of IGFBPs in CRC.

IGFBP	Role	Possible Modes of Action	Reference
IGFBP-1	Protective	Decreased concentration leads to an increased concentration of free IGF-I	[99]
IGFBP-2	Tumor promoting	Local increase in free IGF-I due to proteolysis and binding to α_5_β_1_ integrin	[60,85,86]
IGFBP-3	Protective	Binding of free IGF-I, pro-apoptotic role through NF-κB inhibition, interaction with retinoid X receptor (RXR)/Nur77 or p53-dependent signaling cascade	[79,80,95,96,97]
	Tumor promoting	Undefined mechanisms dependent on other members of the IGF system, mainly the concentrations of IGF-I and IGF-II	[81,82]
IGFBP-4	Protective	Inhibits growth of colon cancer cells	[89,90]
IGFBP-5	Not known	-	-
IGFBP-6	Protective	Inhibition of IGF-II induced proliferation and migration, protection via IGF-independent pathways	[91,92,93,101]
	Tumor promoting	Stimulation of LIM 1215 colon cancer cell migration	[102]
IGFBP-7	Possibly protective	Possibly associated with the activation of apoptosis, but further evidence is required	[108]
